# Regulation of spindle orientation and neural stem cell fate in the *Drosophila *optic lobe

**DOI:** 10.1186/1749-8104-2-1

**Published:** 2007-01-05

**Authors:** Boris Egger, Jason Q Boone, Naomi R Stevens, Andrea H Brand, Chris Q Doe

**Affiliations:** 1 The Wellcome Trust/Cancer Research UK Gurdon Institute and Department of Physiology, Development and Neuroscience, University of Cambridge, Tennis Court Road, Cambridge CB2 1QN, UK; 2 Institute of Molecular Biology, Institute of Neuroscience, Howard Hughes Medical Institute, University of Oregon, Eugene, OR 97403, USA

## Abstract

**Background:**

The choice of a stem cell to divide symmetrically or asymmetrically has profound consequences for development and disease. Unregulated symmetric division promotes tumor formation, whereas inappropriate asymmetric division affects organ morphogenesis. Despite its importance, little is known about how spindle positioning is regulated. In some tissues cell fate appears to dictate the type of cell division, whereas in other tissues it is thought that stochastic variation in spindle position dictates subsequent sibling cell fate.

**Results:**

Here we investigate the relationship between neural progenitor identity and spindle positioning in the *Drosophila *optic lobe. We use molecular markers and live imaging to show that there are two populations of progenitors in the optic lobe: symmetrically dividing neuroepithelial cells and asymmetrically dividing neuroblasts. We use genetically marked single cell clones to show that neuroepithelial cells give rise to neuroblasts. To determine if a change in spindle orientation can trigger a neuroepithelial to neuroblast transition, we force neuroepithelial cells to divide along their apical/basal axis by misexpressing Inscuteable. We find that this does not induce neuroblasts, nor does it promote premature neuronal differentiation.

**Conclusion:**

We show that symmetrically dividing neuroepithelial cells give rise to asymmetrically dividing neuroblasts in the optic lobe, and that regulation of spindle orientation and division symmetry is a consequence of cell type specification, rather than a mechanism for generating cell type diversity.

## Background

The division modes of stem cells are tightly regulated during development and adult tissue homeostasis. This ensures that tissues and organ systems develop to the correct size and contain the correct cell types for proper function. One way to expand the pool of stem or progenitor cells during development is to undergo symmetric cell division. Conversely, one way to generate differentiating cell types, while maintaining a constant stem/progenitor population, is to undergo asymmetric cell division where one daughter differentiates and the other remains a stem cell [1]. Recently, it has been suggested that the ratio of stem/progenitor cells to differentiating cells in a tissue can be regulated by changing spindle orientation, thereby altering the proportion of symmetric to asymmetric cell divisions. For example, it has been proposed that mammalian neuroepithelial cells first expand via symmetric divisions, followed by a burst of neuron production resulting from asymmetric divisions [2]. Recently, it has been reported that altering the division axis in several different vertebrate cell types can lead to a change in fate, for example, in mammalian basal epidermal cells, neural progenitor cells in the developing neocortex and progenitors in the developing retina [3-5]. Despite the recent advances in understanding stem cell self-renewal and spindle orientation in both mammalian and *Drosophila *systems [6], however, very little is known about the relationship between spindle orientation and cell type specification. Do stochastic changes in spindle orientation generate cell diversity during normal development, or does spindle orientation always respond to cell type specification?

In *Drosophila*, the central nervous system is derived from neural stem cells called neuroblasts. There are at least three types of neuroblasts: embryonic, larval central brain/thoracic, and larval optic lobe. They all undergo asymmetric cell division, self-renewing the neuroblast while producing a differentiating daughter cell (ganglion mother cell; GMC). Embryonic neuroblasts delaminate as single cells from a polarized epithelium called the ventral neuroectoderm. Whereas neuroectodermal cells divide symmetrically with a horizontal mitotic spindle (in the plane of the neuroectoderm), neuroblasts rotate their spindles to a vertical plane (perpendicular to the neuroectoderm) and divide asymmetrically to generate a large apical neuroblast and a smaller basal GMC. The GMC typically generates two post-mitotic neurons. Embryonic neuroblast divisions are molecularly and physically asymmetric: the neuroblast inherits apical proteins (for example, atypical Protein kinase C (aPKC) and Inscuteable (Insc)) and the GMC inherits basal proteins (for example, Miranda (Mira), Prospero (Pros), Numb, and Partner of Numb (Pon)) [7]. Larval central brain/thoracic neuroblasts derive from embryonic neuroblasts and undergo a similar asymmetric cell division along their apical/basal axis of polarity. Progress has been made in understanding the molecules that are involved in the self-renewing capacity of larval central brain neuroblasts, and of how misregulation of these factors can lead to tumor formation [8-12]. However, little is known about symmetric divisions in the nervous system and what the molecular switch is that leads to asymmetric division.

In contrast to embryonic neuroblasts and larval central brain neuroblasts, the third class of neuroblasts, those residing in the optic lobe, has been less well characterized. The optic lobe derives from an embryonic optic placode that invaginates into the embryo [13]. The optic lobe cells start to proliferate soon after larval hatching and separate into an outer proliferation centre (OPC) and an inner proliferation centre (IPC). The OPC generates the outer medulla and the lamina neurons; the IPC generates the inner medulla, the lobula and the lobula plate neurons [14]. It has been reported that the early optic lobe cells are neuroblasts that divide symmetrically to expand the population and then later switch to asymmetric division to produce the neurons of the visual system [15,16]. An alternative hypothesis suggests that early optic lobe cells comprise a symmetrically dividing epithelial sheet that later generates asymmetrically dividing neuroblasts by an unknown mechanism [17-19]. However, the lineage relationship between cell types of the optic lobe has never been directly determined, and it is formally possible that the early symmetrically dividing epithelial cells and later developing asymmetrically dividing neuroblasts are two separate cell pools that do not contribute to each other.

Here we use newly available molecular markers, live imaging methods, and genetic lineage techniques to investigate the relationship between symmetrically dividing early progenitors and the asymmetrically dividing neuroblasts of the optic lobe. We test whether changes in spindle orientation are sufficient to induce neuronal differentiation, as has been inferred for the mammalian retina [5]. We find that optic lobe neuroblasts derive from the lateral optic lobe neuroepithelium; that there is a transition from symmetric to asymmetric stem cell-like divisions between these two progenitor populations; and that inducing vertical spindle orientation in neuroepithelial cells is not sufficient to generate ectopic neuroblasts or neurons. Therefore, spindle orientation does not determine cell fate, but is itself regulated in response to cell type specification.

## Results

### Optic lobe morphogenesis

**Figure 1:**
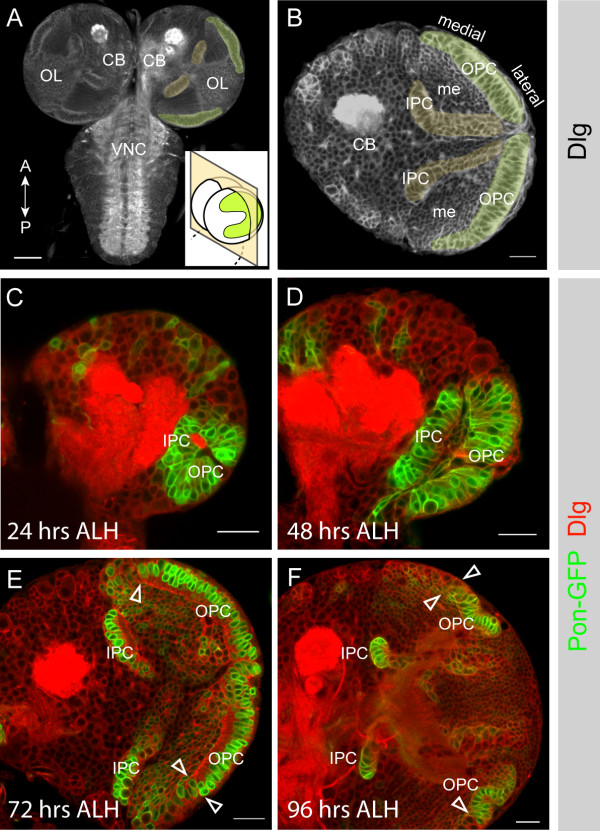
*GAL4*^*c855a *^reveals the proliferation centers of the developing optic lobe. **(a) **A late third instar larval central nervous system (CNS): ventral nerve cord (VNC), central brain (CB) and optic lobes (OL). Subsequent images show frontal confocal sections, as shown in the inset diagram (OPC in green). Anterior and posterior refer to the neuraxis of the larval CNS. **(b) **A frontal section through a brain lobe at mid third instar: the OPC (green), the inner proliferation centre (IPC, yellow) and the medulla cortex (me). Discs large (Dlg; grey) outlines all cell cortices in the larval brain and highlights the morphology of the two optic lobe proliferation centres. **(c) ***GAL4*^*c855a *^begins to drive expression of *UAS-pon-gfp *(green; Dlg in red) at first instar. At late first/early second instar (24 hours ALH; after hatching), the OPC and the IPC can be distinguished as two closely associated epithelia. The cells belonging to the proliferation centers (green) are clearly distinguishable by their columnar shape, in contrast to the round, isolated central brain cells. **(d) **At the end of second/early third instar (48 hours ALH) the epithelia of the OPC and IPC separate from each other and smaller progeny cells are located between the two epithelia. **(e) **As development progresses during second to mid third instar (72 hours ALH) the OPC cells at the medial edge of the epithelium loose their columnar shape (to the left of the arrowheads). **(f) **At late third instar (96 hours ALH) the OPC epithelium decreases in size while the number of round neuroblast-like cells increases at the medial edges (to the left of the arrowheads). All images are single confocal sections, with anterior on top and lateral to the right. Scale bar is 50 μm (a) and 20 μm (b-f).

We screened a collection of GAL4 enhancer trap lines to identify markers for optic lobe cell types. The expression of one line, *GAL4*^*c855a *^[20,21], is restricted to the optic lobes (Figure 1). We used this line to drive expression of *UAS-partner of numb-gfp *(*pon-gfp*) [22] and followed optic lobe morphogenesis throughout larval development (Figure 1). Frontal brain confocal sections show that, at mid third instar, the developing OPC of the optic lobe forms a dome-shaped shell covering the lateral brain lobe with an opening pore at its center, while the IPC is U-shaped with the opening of the U pointing in the dorso-caudal direction (Figure 1a, b) [18]. This structure arises from a small group of 30 to 40 progenitor cells in newly hatched larvae [18]; by 24 hours after larval hatching (ALH) the OPC and the IPC can be distinguished (Figure 1c) and each population forms an expanding epithelial sheet throughout larval development (Figure 1d–f). By the second instar larval stage, a population of cells at the medial edges of the OPC epithelium appears to round up, loses epithelial morphology, and down-regulates *GAL4*^*c855a*^. These are likely to be the previously described OPC neuroblasts [18,19].

### The optic lobe consists of two distinct cell types

Previous studies have drawn different conclusions about the cell types of the optic lobe. Some reports suggest that the early optic lobe consists initially of symmetrically dividing neuroblasts that, at later stages, become asymmetrically dividing neuroblasts [15,16]. In contrast, other reports conclude that the early optic lobe consists of epithelial cells and only later do neuroblasts develop at the medial edges of the epithelium [18,19]. In the latter studies it has been assumed that the optic lobe neuroblasts derive from the optic lobe epithelium, but this has never been tested directly by lineage studies. In this section and the following one, we discuss the use of molecular markers, live imaging experiments, and genetic cell lineage analysis to resolve the identity and origins of these optic lobe cell types.

**Figure 2:**
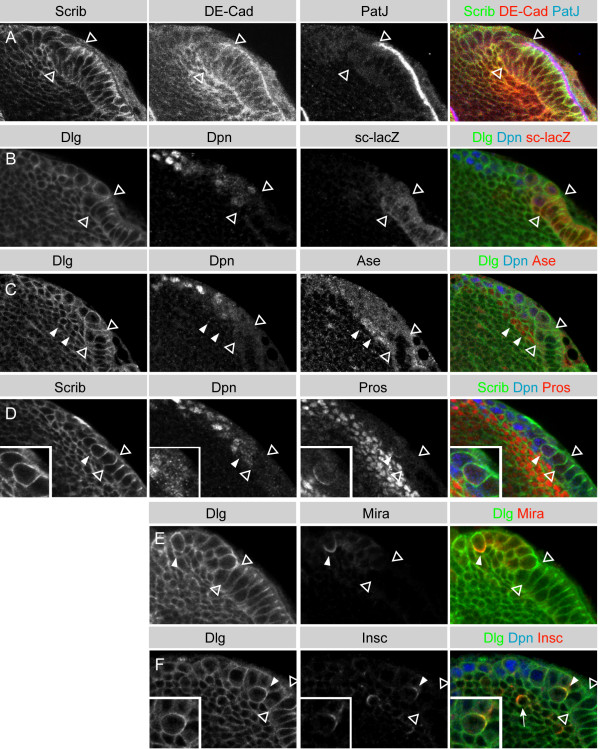
Optic lobe neuroepithelial cells and neuroblasts are arranged in distinct medio-lateral zones. **(a) **The developing optic lobe generates a lateral epithelial region (to the right of the arrowheads). Epithelial cells express three proteins that localize to cellular junctions: Scrib (green) localizes to the basolateral septate junctions in epithelial cells; DE-Cad (red) localizes to the basolateral zonula adherens; and PatJ (blue) localizes to apical and subapical regions in epithelial cells. Medial neuroblasts (to the left of the arrowheads) are more round and lack the clear subcellular localization of these junctional proteins. **(b) ***sc-lacZ *(red) is expressed in the lateral epithelium of the optic lobe (to the right of the arrowheads). Expression is diminished in medial optic lobe neuroblasts (to the left of the arrowheads). Medial neuroblasts express the bHLH transcription factor Dpn (blue), which is not expressed by neuroepithelial cells. Dlg (green) outlines all cell cortices but is enriched at adherens junctions. **(c) **Asense (red) shows weak cytoplasmic expression in medial Dpn (blue) positive neuroblasts (to the left of the open arrowheads). Asense is nuclear in the progeny of neuroblasts (filled arrowheads). **(d) **Pros protein (red) forms a basal crescent (inset) in mitotic medial optic lobe neuroblasts (filled arrowhead). Dpn (blue) is restricted to the neuroblasts but Pros (red) is inherited by the basal progeny cells where it localizes to the nucleus. **(e) **Mira (red) forms a basal crescent in mitotic neuroblasts (filled arrowhead) (metaphase; n = 9 and telophase n = 9). Note that neither Pros nor Mira are present in neuroepithelial cells (to the right of the arrowheads). **(f) **Insc protein (red) forms an apical crescent in mitotic medial optic lobe neuroblasts (filled arrowhead and inset). These neuroblasts reveal weak cytoplasmic Dpn (blue). Dlg (green) is enriched apically, where it co-localizes with the Insc crescent at the apical cortex (inset). Some progeny cells in the medulla cortex also express *insc *(arrow). All images are single confocal sections from third instar brains, with anterior to the top and lateral to the right. Open arrowheads mark the boundary between the neuroepithelium (to the right) and the neuroblast zone (to the left).

We first tested whether the optic lobe contains epithelial cells by staining for epithelial junctional marker proteins. PatJ is a cytoplasmic scaffolding protein and is part of the conserved Crumbs complex, which is located in apical and subapical regions in epithelial cells. DE-Cadherin (DE-cad) is a transmembrane protein located at the zonula adherens, while Discs large (Dlg) and Scribble (Scrib) are PDZ domain tumor suppressor proteins that are enriched at the basolateral septate junctions [23]. We found that a subpopulation of the optic lobe cells, those that express *GAL4*^*c855a *^and have epithelial morphology (Figure 1), express all of these junctional markers, and that they localize to their appropriate cellular domains (Figure 2a). Thus, the optic lobe contains an epithelial cell population that expands during early larval stages and becomes depleted by pupariation (Figure 1).

To determine if these epithelial cells have neuroepithelial features, we assayed for the expression of the proneural genes *achaete *(*ac*) and *scute *(*sc*). *ac *and *sc *are expressed in clusters of cells in other epithelia (for example, embryonic ventral ectoderm and imaginal discs) where they promote neurogenesis. Delta-Notch signaling antagonizes proneural expression, resulting in only one or a few cells in the cluster developing as a neuroblast (embryo) or a sense organ precursor (imaginal disc), while the remaining cells adopt an epidermal fate [24,25]. We found that all cells in the OPC express the proneural gene *scute *(Figure 2b; Additional data file 1), but we observed no expression of the proneural gene *achaete *(data not shown). Thus, the optic lobe epithelium is a neuroepithelium and all cells in the epithelial sheet appear to have the potential to enter the neural pathway.

We next assayed neuroblast markers, to determine if the neuroepithelial cells are actually neuroblasts undergoing symmetric divisions to expand the neuroblast population [15,16]. We stained for Deadpan (Dpn) and Mira, which label all known embryonic and larval central brain neuroblasts [9-11,26,27] and found that these markers failed to label the neuroepithelial cells of the optic lobe (Figure 2b–e). They did, however, label a population of rounded cells at the edge of the epithelium, which lacked Dlg/Scrib septate junction localization (Figure 2b–d) and were positioned at the site of the previously described optic lobe neuroblasts [18,19]. This neuroblast population is closely associated with strings of smaller cells that express the GMC markers nuclear Pros and nuclear Asense (Ase) (Figure 2c, d). Lineage analysis, described below, confirmed that these smaller Pros+ cells are neuroblast progeny. Thus, based on molecular markers and morphology, we detected two distinct populations of cells in the developing optic lobe: neuroepithelial cells and neuroblasts. We found no evidence of a population of symmetrically dividing neuroblasts in the optic lobe.

### Optic lobe neuroepithelial cells divide symmetrically, whereas neuroblasts divide asymmetrically

**Figure 3:**
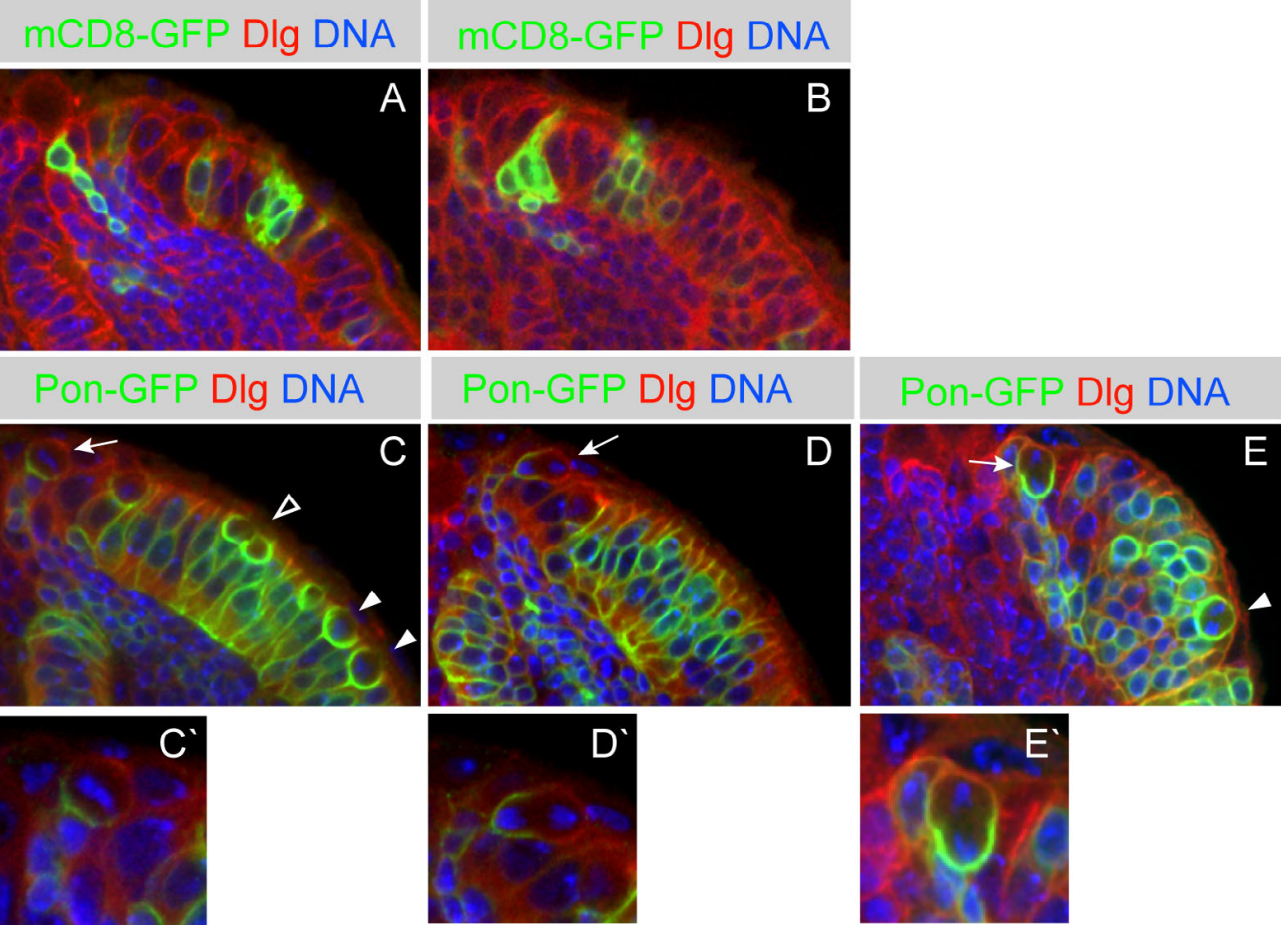
Proliferative symmetric and differentiative asymmetric division depends on the medio-lateral location within the optic lobe. **(a, b) **mCD8-GFP MARCM clones (green) are shown in mid third instar brains. Dlg is in red and DNA in blue. **(a) **A lateral clone contains columnar shaped epithelial cells that presumably were generated by proliferative symmetric divisions (the single confocal section shows three epithelial cells). The clone located at the medial edge of the optic lobe contains neuroblasts with attached strings of progeny cells (the single confocal section shows one neuroblast and three progeny cells). **(b) **A clone at the medial edge of the optic lobe comprises four progenitor cells and one progeny cell (the single confocal section shows two progenitor cells and one progeny cell). **(c-e) ***GAL4*^*c855a *^driven *UAS-pon-gfp *(green) reveals the division mode of optic lobe neuroepithelial cells and neuroblasts. Dlg is in red and DNA in blue. Brains at mid-third (c, d) and early third (e) instar. **(c) **Neuroepithelial cells undergoing mitosis round up at the apical surface of the epithelium and show basolateral Pon-GFP (metaphase: filled arrowheads). Upon cytokinesis Pon-GFP is partitioned equally to both daughter cells (telophase: open arrowhead). At the medial edge of the epithelium optic lobe neuroblasts reveal a basal crescent of Pon-GFP at metaphase (arrow; enlarged in (c')). **(d) **At the medial edge of the epithelium a neuroblast in anaphase segregates Pon-GFP asymmetrically to the basal daughter cell (arrow; enlarged in (d')). **(e) **A more dorsal confocal section reveals a neuroepithelial cell in anaphase segregating Pon-GFP symmetrically to both daughter cells (arrowhead) and a neuroblast (arrow; enlarged in (e')) in anaphase segregating Pon-GFP to the basal daughter cell. All images are single confocal sections, with anterior on top and lateral to the right.

To test our conclusion that neuroepithelial cells divide symmetrically and neuroblasts divide asymmetrically, we assesed the localization of cortical polarity proteins in the optic lobe by immunohistochemistry and live imaging. Insc and aPKC localize to the apical cortex of embryonic and larval neuroblasts [28-30], whereas Mira, Pros, and Pon-GFP are basally localized in some epithelial cells and in all neuroblasts [27,31-34]. We found that Dpn-positive optic lobe neuroblasts always segregate Insc (Figure 2f) and aPKC (data not shown) into the larger neuroblast and Mira, Pros, and Pon-GFP into the smaller GMC (n = 37; (Figures 2d, e, 3c–e; Additional data files 2 and 3). In contrast, most Dpn-negative neuroepithelial cells partition Pon-GFP equally to both daughter cells (n = 28) (Figure 3c, e; Additional data file 4) and we did not detect expression of Insc, Mira or Pros. The only exception is a population of Dpn-negative epithelial cells that lie adjacent to the Dpn-positive neuroblasts, which segregate Pon-GFP asymmetrically. These cells are likely to be newly formed neuroblasts with Dpn levels below our detection threshold.

To further characterize the neuroepithelial and neuroblast populations in the optic lobe, we next investigated their cell division patterns in wild-type brains. We used the MARCM system [35] to induce small mCD8-GFP labeled wild-type clones at late second/early third larval instar (48 hours ALH) and analyzed the brains at mid-third instar (72 hours ALH) (Figure 3a). We observed small clones containing two to eight cells with columnar epithelial morphology (n = 7) within the lateral optic lobe, consistent with the expansion of one progenitor via symmetric cell division (Figure 3a). We also saw clones in the medial optic lobe (where the neuroblasts are located) that had one or more large round cells adjacent to a cluster of smaller round cells (n = 11), consistent with neuroblasts dividing asymmetrically to generate a chain of smaller GMCs/neurons (Figure 3a). We conclude that neuroepithelial cells divide symmetrically to generate two neuroepithelial cells, whereas neuroblasts divide asymmetrically to generate smaller progeny.

The combination of our molecular, morphological, and live imaging data allows us to conclude that there are two distinct cell types in the optic lobe. Neuroepithelial cells are found in the lateral region and have a classic columnar epithelial morphology, epithelial molecular markers and epithelial junctions. They undergo symmetric cell division to expand the neural stem cell population. Neuroblasts are found in the medial region and have a rounded shape and lack epithelial junctions. They divide asymmetrically to self-renew and produce a smaller differentiating daughter cell.

### Optic lobe neuroepithelial cells are the progenitors of optic lobe neuroblasts

**Figure 4:**
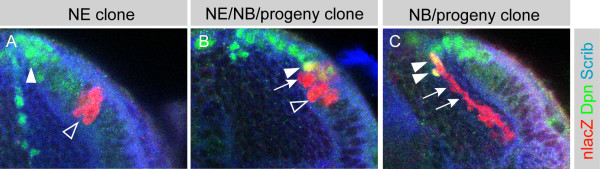
Optic lobe neuroblasts derive from the neuroepithelium in a medial transition zone. **(a-c) **Single FLP-out clones expressing nuclear β-gal (red) in the optic lobe at late third instar (96 hours ALH). Dpn is in green, Scrib in blue. **(a) **An epi only clone containing Dpn negative epithelial cells (marked with β-gal in red, open arrowhead) but no Dpn positive neuroblasts (green, arrowhead). **(b) **An epi/NBs/progeny clone containing Dpn negative epithelial cells (open arrowhead), Dpn positive neuroblasts (arrowhead, yellow) and progeny cells (arrow). **(c) **A NBs/progeny clone containing Dpn positive neuroblasts (arrowheads, yellow) and progeny cells (arrow).

We next wished to test directly the hypothesis that optic lobe epithelia give rise to optic lobe neuroblasts [17-19]. We performed a clonal analysis using the FLP/FRT system [36] and adjusted clone induction frequency to 1.2 clones per optic lobe. We induced clones expressing a nuclear β-galactosidase (β-gal) reporter protein at early second instar (31 hours ALH), when the optic lobe consists primarily of neuroepithelial cells (Figure 1), and assayed the developing clones for cell fate markers at 48 hours or 96 hours ALH. Brains were labeled for β-gal to show all cells within a clone; for Scrib to outline cell morphology and label epithelial septate junctions; and for Dpn to mark neuroblasts (Figure 4). We observed four classes of clones: neuroepithelial cells only (Figure 4a); neuroblasts and their neuronal progeny only (Figure 4c); neuronal progeny only (data not shown); and mixed clones of neuroepithelial cells, neuroblasts and progeny (Figure 4b). When clones were assayed relatively soon after induction (48 hours ALH), we observed a high percentage of neuroepithelial only clones (22/28), with few neuroblast only clones (5/28) or mixed clones (1/28). In contrast, allowing the clones to develop longer (96 hours ALH) resulted in a majority of the clones being neuroblast/progeny only (20/33) or neuronal progeny only (4/33), with few neuroepithelial only clones (5/33) or mixed clones (4/33).

One example of a clone that supports the idea of a switch from a neuroepithelial to neuroblast cell type is shown in Figure 3b. This clone, at the medial edge of the epithelium, contains four neural progenitor cells and one progeny cell, suggesting that a neuroepithelial cell underwent two rounds of symmetric division to generate four cells; one of these cells then switched to a neuroblast fate and divided asymmetrically, self-renewing and producing a single GMC. Another such clone consisted of 20 neuroepithelial cells, four large round Dpn positive cells, and two smaller round cells (data not shown). We interpret this clone as deriving from a neuroepithelial cell that divided symmetrically to generate 24 cells, four of which switched to a neuroblast fate. Two of these neuroblasts then divided asymmetrically to produce a single GMC each.

Two conclusions can be drawn from our lineage experiments. First, neuroepithelial cells give rise to neuroblasts; initially most clones consist exclusively of neuroepithelial cells but with time most clones contain neuroblasts and their progeny. It is likely that neuroepithelial clones that expand towards the medial edge of the epithelium become partially or completely transformed into neuroblasts. This is consistent both with previous studies and our own observations that the epithelial population shrinks as the neuroblast population expands (Figure 1) [18,19]. Second, at least some neuroblasts ultimately differentiate or die, resulting in clones that consist entirely of neuronal progeny.

### Inducing vertical spindle orientation in neuroepithelial cells does not promote neuroblast or neuronal specification

It has been proposed that mammalian neuroepithelial cells, retinal progenitor cells and epidermal stem cells expand their stem cell population by 'horizontal' divisions in which the mitotic spindle aligns perpendicular to the apical/basal axis of cell polarity. They then switch to a 'vertical' division axis to divide asymmetrically and generate novel cell types [2-5,37,38]. It is not known whether a change in cell fate is required to switch the cell division axis (for example, to a cell fate that expresses a protein that modifies spindle orientation), or whether a stochastic change in spindle orientation can lead to a cell fate change (for example, due to the asymmetric partitioning of cell fate determinants). The *Drosophila *optic lobe neuroepithelium represents an excellent model system to determine whether a change in spindle orientation induces new cell fates, or whether a change in cell fate is required to alter spindle orientation.

**Figure 5:**
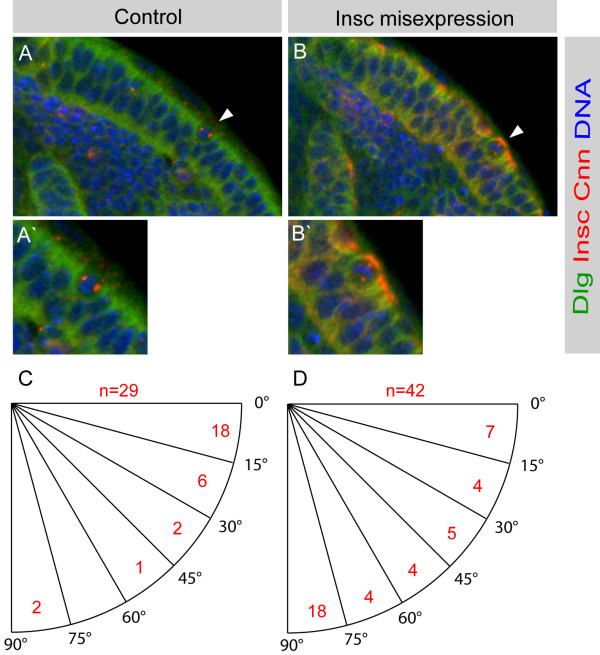
Misexpression of Insc in neuroepithelial cells can induce vertical spindle orientation. Spindle orientation at prometaphase/metaphase was analyzed in neuroepithelial cells at mid third instar (72 hours ALH). **(a) **In control brains the great majority of neuroepithelial cells have a horizontal spindle axis (arrowhead, enlarged in (a')) (n = 29). Note that neuroepithelial cells do not express Insc. **(b)***GAL4*^*c855a *^driven *UAS-insc *results in apical Insc in neuroepithelial cells and forces spindles into a vertical orientation (n = 42). **(c, d) **Spindle orientation in control optic lobes (c) and optic lobes misexpressing Insc (d). A horizontal spindle axis is 0°; a vertical spindle axis is 90°. The number of neuroepithelial cells is shown in red within six 15° angle sectors from 0° to 90°.

To switch spindle orientation in neuroepithelial cells we misexpressed Insc in neuroepithelial cells. Expression of Insc in embryonic epithelial cells has been shown to reorient their mitotic spindles from horizontal (perpendicular to the apicobasal axis) to vertical (aligned with the apicobasal axis) [30]. Embryonic Insc misexpression does not lead to obvious changes in the embryonic neuroectoderm. However, not all neuroectodermal cells give rise to neural precursors; most give rise to epidermis. In the optic lobe, all neuroepithelial cells express the proneural gene *sc *and are, therefore, competent to become neuroblasts. Therefore, we investigated whether spindle reorientation can induce a neuroblast fate in this system. In control optic lobe neuroepithelia we detected no Insc protein and the majority of metaphase spindles were aligned horizontally, positioned to give a symmetric cell division (Figure 5a, c). When Insc is misexpressed within the optic lobe neuroepithelium, the protein localizes apically and the majority of metaphase spindles orients vertically, along the apicobasal axis, positioned to enable an asymmetric cell division (Figure 5b, d). Despite this striking reorientation of the mitotic spindle, we saw no evidence for the induction of ectopic Dpn+ neuroblasts, GMCs, or neurons in the optic lobe (data not shown). We conclude that forcing vertical spindle orientation in neuroepithelial cells is not sufficient to induce neuroblast or GMC cell fates. After Insc misexpression the neuroepithelium is virtually indistinguishable from a control neuroepithelium throughout larval development. We conclude that the resulting apical and basal daughter cells are reintegrated into the epithelium and are only able to switch to a neuroblast fate when they reach the edge of the optic lobe. Thus, the transition from neuroepithelial cell to neuroblast must be due to a cell fate transition that is not regulated by a switch in spindle orientation. We propose that the switch from a neuroepithelial cell to a neuroblast entails the coordinate regulation of multiple downstream events that include the disassembly of epithelial junctions and the transcription of genes that promote vertical spindle orientation.

## Discussion

**Figure 6:**
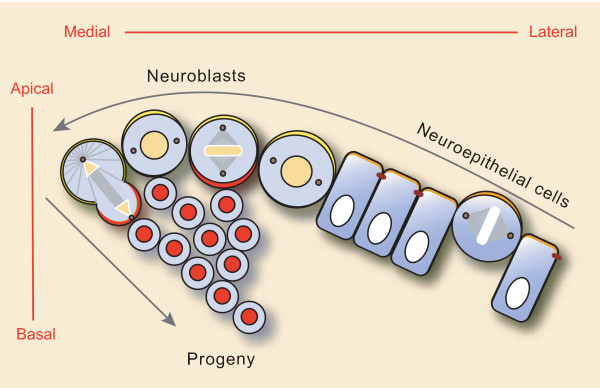
Model of neuroepithelial to neuroblast transition at the medial edge of the optic lobe. At the medial edge of the optic lobe columnar neuroepithelial cells disassemble adherens junctions and undergo a transition to neuroblasts. Neuroepithelial cells divide symmetrically with horizontal spindle orientation, which results in the expansion of the progenitor pool. Medial neuroblasts divide asymmetrically with vertical spindle orientation and bud off smaller ganglion mother cells (GCMs) towards the presumptive medulla cortex.

In this study we show that optic lobe neuroepithelial cells can be distinguished from optic lobe neuroblast cells by morphology, gene expression and division mode (Figure 6). Neuroepithelial cells occupy the lateral region of the optic lobe and divide in a proliferative symmetric division mode, which expands the neural stem cell pool at an early phase of optic lobe development. At a later stage, progressively more stem cells round up and split off from the medial part of the optic lobe epithelium. These optic lobe neuroblasts lose their adherens junctions and start to divide asymmetrically, generating smaller GMCs towards the growing medulla cortex.

The optic lobe neuroepithelium is similar to the embryonic ventral neuroectoderm in that it expresses the same junctional complexes and the proneural gene *scute*. Optic lobe neuroblasts exhibit an apicobasal polarity and express pan-neural genes such as *dpn *and *ase*. However, most embryonic neuroectodermal cells adopt an epidermal fate, whereas optic lobe epithelial cells eventually give rise to neuronal and glial cells (hence it is a neuroepithelium). Recently, it has been suggested that embryonic neuroblasts require an extrinsic signal, provided by the overlying epithelium, to coordinate their division axis with apicobasal tissue polarity [39]. As optic lobe neuroblasts do not delaminate from an overlying (apical) epithelium, but rather segregate laterally from the adjacent neuroepithelium, they do not maintain contact with an overlying epithelium. Nonetheless, they are still able to reorient their mitotic spindles and divide asymmetrically along the apicobasal axis, budding off GMCs towards the developing medulla cortex. The cortex glial cells, which enwrap the larval brain [40], may provide apicobasal positional information to the optic lobe neuroblasts in place of an overlying epithelium.

In the *Drosophila *embryo the proneural genes *ac*, *sc*, and *lethal of scute *are expressed in the neuroectoderm [41,42], as is the transcription factor Pros and its adaptor Mira [32-34]. Although we saw proneural gene expression in the optic lobe neuroepithelium, we detected neither Pros protein nor Mira mRNA or protein. This contrasts with the embryonic neuroectoderm, where both Pros and Mira are expressed and localize basolaterally, and suggests that the transcriptional cascade underlying optic lobe neuroblast formation is different from embryonic neuroblast formation. In the optic lobe, Mira and Pros are first expressed in neuroblasts. Here they localize in a crescent at the basal cortex and segregate into the medulla GMCs (Figure 2d, e) (in contradiction to an earlier study suggesting that Pros is not expressed in optic lobe neuroblasts and GMCs, but only in mature neurons [16]).

### Possible mechanisms for the transition from optic lobe neuroepithelial cells to neuroblasts

Our clonal analysis demonstrates that optic lobe neuroblasts derive from the optic lobe neuroepithelium in a temporally and spatially regulated fashion. In assessing the clonal relationship between optic lobe neuroepithelial cells and neuroblasts we recovered only a small number of mixed clones containing both epithelial cells and neuroblasts. Instead, most clones contained either only epithelial cells or neuroblasts and their progeny. The transition from a neuroepithelium to neuroblasts could occur by a neuroepithelial cell dividing symmetrically, generating two neuroblasts, or by a neuroepithelial cell dividing asymmetrically, generating one neuroepithelial cell and one neuroblast. Our clonal analysis does not distinguish whether one or both of these mechanisms occur.

A mediolateral gradient of a morphogen may regulate the changes in gene expression required to induce the neuroblast fate. Once the neuroepithelium has proliferated to reach a critical size, the most medial cells would be pushed beyond the range of the morphogen's activity, and would be induced to become neuroblasts. A possible candidate for this morphogen is Decapentaplegic (Dpp), the *Drosophila *BMP2/4 homologue, which shows regional, Wingless-dependent, expression in the optic lobe [43]. Mutations in either *wg *or *dpp *lead to a reduction in the size of the optic lobe and to defects in the optic lobe neuropile and it has been suggested that these defects might be caused by failure in progenitor specification in the developing optic lobe [43].

### Similarities to vertebrate neural stem cells

The transition of optic lobe neuroepithelial cells to neuroblasts in the optic lobe is reminiscent of the transition of neuroepithelial cells to radial glia in the developing vertebrate neocortex and in the neural tube. Mammalian neuroepithelial cells, or neural stem cells, first undergo symmetric division to expand the neural stem cell pool. This is followed by self-renewing, asymmetric division, during which neuroepithelial cells down-regulate epithelial features such as tight junctions (but not adherens junctions) and self renew while also generating cells with a more restricted developmental potential [44-50].

The organization of the optic lobe also bears comparison with the vertebrate retina, where a spatially ordered structure is evident with respect to both cellular development and differentiation: in the ciliary marginal zone (CMZ) the youngest and least determined stem cells are closest to the periphery, the proliferative retinoblasts are medial and the cells that have stopped dividing are at the central edge [51,52]. Similarly in the optic lobe, the neuroepithelial cells are found laterally, the neuroblasts medially, and the ganglion cells towards the inside of the lobe.

The striking similarities between the optic lobe and the CMZ suggest that similar genetic pathways may be involved in both systems. Recently, it was shown that Insc, which regulates spindle orientation in *Drosophila *neuroblasts, is also expressed in the vertebrate retina [5]. Insc expression in embryonic neuroblasts and optic lobe neuroblasts is one of the earliest signs of neuroblast specification; neither the embryonic ventral neuroectoderm nor the optic lobe neuroepithelium express *insc*. Interestingly, whereas *insc *is expressed in vertically dividing neuroblasts in the *Drosophila *optic lobe and embryonic central nervous system, in the mammalian retina it is expressed in both vertically dividing cells (where it localizes apically) and horizontally dividing cells (where it is apicolateral). This suggests that, in the vertebrate retina, the division plane is determined by whatever localizes Insc, rather than solely by the presence of Insc.

Zigman *et al*. [5] show that reducing the levels of Insc increases the number of horizontal divisions at the expense of vertical divisions. This leads eventually to a decrease in the number of early differentiating photoreceptor cells and eventually to an increase in later differentiating bipolar neurons. From these results the authors infer that a switch from vertical to horizontal division increases the stem cell pool at the expense of early differentiated neurons, that is, that spindle orientation determines the fate of the progenitor cells.

## Conclusion

Here we show that the optic lobe harbors two neural stem cell types: neuroepithelial cells, which divide symmetrically to expand the neural stem cell pool, and neuroblasts, which divide asymmetrically to self-renew and generate differentiating GMCs. Neuroblasts derive from the neuroepithelium in a developmentally and spatially regulated fashion. Reorientation of the mitotic spindle in *Drosophila *neuroepithelial cells, as directed by ectopic expression of Insc, is not sufficient in and of itself to induce the neuroblast fate and does not lead to premature neurogenesis. Instead, spindle orientation responds to cell fate rather than promoting it. Cell fate specification in neuroblasts leads to expression of *insc *and spindle reorientation. A second consequence of neuroblast fate specification is the expression of Pros and Mira. Thus, when the spindle reorients in the neuroblast, cell division generates two different cell types due to the asymmetric partitioning of Pros. In the optic lobe the different division planes of neuroepithelial cells and neuroblasts lead to stratified layers of cells that contribute to the morphogenesis of the brain lobes (Figure 6). Thus, one key role of regulated spindle orientation in the optic lobe may be in positioning cells appropriately within the tissue, a function similar to what has been proposed for mammalian skin [3].

## Materials and methods

### Fly strains

Flies were raised on cornmeal medium at 25°C. Oregon R and *yw *were used as control strains. To assay *sc *expression the *3.7sc-lacZ *line [53] (from P Simpson, Cambridge, UK) was used. The following driver and responder lines were used: *GAL4*^*c855a *^[20,21] (from the Bloomington *Drosophila *Stock Centre, Bloomington, Indiana, USA), *UAS-pon-gfp *[31], *UAS-pon-gfp; UAS-H2B-mRFP1 *[54] (from Y Bellaiche, Paris, France) and *UAS-insc/TM3 *[30] (from J Knoblich, Vienna, Austria). For MARCM clones we used *hs-Flp; FRT40A, tub-Gal80; tub-Gal4/TM6B *and *FRT40A; UAS-mCD8-GFP, UAS-nlslacZ *[8] (from B Bello, Basel, Switzerland). For flip-out clones and lineage tracing *hs-FLP(f38) *and *act5C(FRT)nlslacZ *(from Bloomington) were used.

### Staging of larvae and clone induction

Freshly hatched larvae were collected in a 4 to 6 hour time window and staged on cornmeal medium to late first/early second instar (21 to 27 hours ALH; after hatching), late second/early third instar (45 to 51 hours ALH), mid third instar (69 to 75 hours ALH) or late third instar (93 to 99 hours ALH). Targeted gene expression was achieved with the GAL4/UAS system. The *GAL4*^*c855a *^line drives targeted gene expression in all optic lobe progenitor cells from first instar onwards. For MARCM experiments clones were induced by heat shock for 30 minutes at 37°C at late second/early third instar with the following genotype: *yw, hs-FLP; FRT40A, +/FRT40A, tub-GAL80; UAS-mCD8:GFP, UAS-nlslacZ/tub-GAL4*. Larvae were dissected and fixed at mid third instar for clone examination. For flip-out clonal analysis clones were induced by heat-shock for 45 minutes at 37°C at 31 hours ALH. Clones were examined at 48 hours or 96 hours ALH.

### Insc misexpression and analysis of spindle axis

For insc misexpression *GAL4*^*c855a *^was crossed to UAS-insc/TM3. The spindle axis was analyzed in *GAL4*^*c855a*^*/UAS-insc *and *GAL4*^*c855a*^*/TM3 *control brains. For cells in prometaphase and metaphase a line was drawn joining the two centrosomes. The angle of the spindle axis was calculated in reference to the tangent at the neuroepithelial surface. We only considered Dpn negative cells that were within the neuroepithelium and not neighboring Dpn positive neuroblast regions.

### Immunocytochemistry and image acquisition

Larval tissues were fixed and immunostained as previously decribed in [55]. Primary antibodies used in this study include rabbit anti-Scrib 1:2500 [56], rat anti-DE-Cad 1:100 (Serotec, Raleigh, North Carolina, USA), rabbit anti-PatJ 1:1000 [57] (renamed PatJ [58]), mouse anti-Dlg 4F3 1:100 (Developmental Studies Hybridoma Bank (DSHB), Iowa City, Iowa, USA), rat anti-Dpn 1:2 [10], rabbit anti-Ase 1:500 (from A Jarman, Edingburgh, UK), mouse anti-Pros MR1A (DHSB) 1:30, rabbit anti-Mira A96c 1:1000 [33] (from YN Jan, San Francisco, USA), rabbit anti-Insc 1:500 (from W Chia, Singapore, Singapore) mouse anti-βGAL 1:500 (Promega, Madison, Wisconsin, USA), rabbit anti betaGal 1:10000 (Cappel, Organon Teknika Corporation, West Chester, Pennsylvania, USA), rabbit anti-Cnn 1:1000 (unpublished, kindly provided by J Raff, Cambridge, UK), rabbit anti-GFP 1:1000 (Abcam, Cambridge, Cambridgeshire, UK), and chicken anti-GFP 1:20 (Upstate, Charlottesville, Virginia, USA). DNA was stained with DAPI (Sigma-Aldrich, Gillingham, Dorset, UK). Fluorescent conjugated secondary antibodies Alexa405, Alexa488, Alexa568, Alexa633 were used (Molecular Probes, Invitrogen, Paisley, Renfrewshire, UK). Images were acquired with a Leica SP2 confocal microscope and processed with Imaris 3.2 (Bitplane, Zurich, Switzerland) and Adobe Photoshop 8.0. Figures and illustrations were made using Adobe Illustrator 11.0.

### Live imaging

Larval brains expressing *GAL4*^*c855a *^driving Pon-GFP and H2B-mRFP1 were dissected at third instar and placed on poly-Lysine (0.002%) coated coverslips in a chamber containing fat body conditioned D22 insect medium, 7.5% bovine calf serum [59]. Cell divisions were imaged using a Zeiss Meta510 inverted confocal microscope equipped with a 40 × NA 1.4 oil-immersion objective.

### Fluorescent in situ hybridization

Probes were made by using PCR amplification from a cDNA library with the reverse primer containing a T7 polymerase promoter, CAGTAATACGACTCACTATTA. PCR was performed using Phusion Taq (New England Biolabs, Hitchin, Hertfordshire, UK) with the following cycles: 98°C for 2 minutes; 5 times (98°C for 20 s, 50°C for 20 s, 72°C for 1 minute); 35 times (98°C for 20 s, 59°C for 20 s, 72°C for 2 minutes); and 72°C for 5 minutes. The primers were designed using Primer3 [60] with an optimum length of 24 base-pairs (bp) and optimum melting temperature (Tm) of 60°C. UTP-Dig (Roche Diagnostics, Burgess Hill, West Sussex, UK) labeled RNA probes were generated from template PCR products by *in vitro *transcription. For better tissue penetration the probes were degraded to an average size of 500 bp fragments using a carbonate fragmentation buffer [61]. Fluorescent *in situ *hybridization (FISH) was performed according to [62]  with minor modifications. Larval brains were fixed in 4% paraformaldehyde in phosphate-buffered saline for 20 minutes. Hybridization was performed at 65°C for 12 to 16 hours. Fluorescent signal was obtain by using a Tyramide Amplification Kit (Molecular Probes, Invitrogen). Primers for probes were: *ac*_forward, GAAAATCACTCTGTTTTCAACGAC; *ac*_reverse, **CAGTAATACGACTCACTATTA**TCAGTTTAATGTCCTCAATGTATGC; *sc*_forward, ACAACGAAAAGCACTACCATGTCA; *sc*_reverse, **CAGTAATACGACTCACTATTA**AGAAAATAGGGCGTGGTGGTAAAT; *mira*_forward, GGTAGAGAATCTCCAGAAGACCAA; *mira*_reverse, **CAGTAATACGACTCACTATTA**AAACGCGAAAGATAGAAAACAATC. The nucleotides in bold represent the T7 polymerase binding site.

## Competing interests

The author(s) declare that they have no competing interests.

## Authors' contributions

BE participated in the expression studies, carried out the Pon-GFP live study, the MARCM study, the Insc misexpression study and drafted the manuscript. JQB carried out the FLP-out clonal analysis and participated in the expression study; he also helped in drafting the manuscript. NRS participated in designing and performing the Insc misexpression study. AHB and CQD conceived of the study and participated in its design and coordination and drafted the final manuscript. All authors read and approved the final manuscript.

## Supplementary Material

Additional data file 1 *scute *and *mira *mRNA expression in the optic lobe. **(a) **FISH to detect *sc *mRNA (green) in late third instar in combination with immuno-staining for Dlg (red). *sc *mRNA is expressed in the entire epithelium and in the medial neuroblasts. The *3.7sc-lacZ *reporter line (Figure 2b) is expressed strongly in the neuroepithelium and is downregulated in medial neuroblasts. *3.7sc-lacZ *may not reproduce the entire *sc *expression pattern, or *lacZ *expression in medial neuroblasts may be below our detection level. **(b) **FISH for *mira *mRNA (green) in late third instar in combination with immuno-staining for Dlg (red). *mira *mRNA is expressed in medial neuroblasts but not in neuroepithelial cells. Click here for file

Additional data file 2 Optic lobe neuroblasts divide asymmetrically. Asymmetric division of a medial OPC neuroblast in a late third instar brain. *GAL4*^*c855a *^drives Pon-GFP and H2B-mRFP1 in the OPC progenitor cells. Click here for file

Additional data file 3 Unequal segregation of Pon-GFP to the GMC daughter cell. Asymmetric division of an OPC neuroblast in a late third instar brain. *GAL4*^*c855a *^drives Pon-GFP and H2B-mRFP1 in the OPC progenitor cells. Note that Pon-GFP is asymmetrically segregated to the basal daughter cell Click here for file

Additional data file 4 Optic lobe neuroepithelial cells divide symmetrically. Symmetric division of an OPC neuroepithelial cell in late third instar. *GAL4*^*c855a *^drives Pon-GFP and H2B-mRFP1 in OPC progenitor cells. Note that Pon-GFP is symmetrically segregated to both daughter cells Click here for file
