# Supplementary figures and images for: Regulation of spindle orientation and neural stem cell fate in the Drosophila optic lobe
Source: Neural Dev. 2007 Jan 5;2:1. doi: 10.1186/1749-8104-2-1 (PMC1779784; doi:10.1186/1749-8104-2-1)

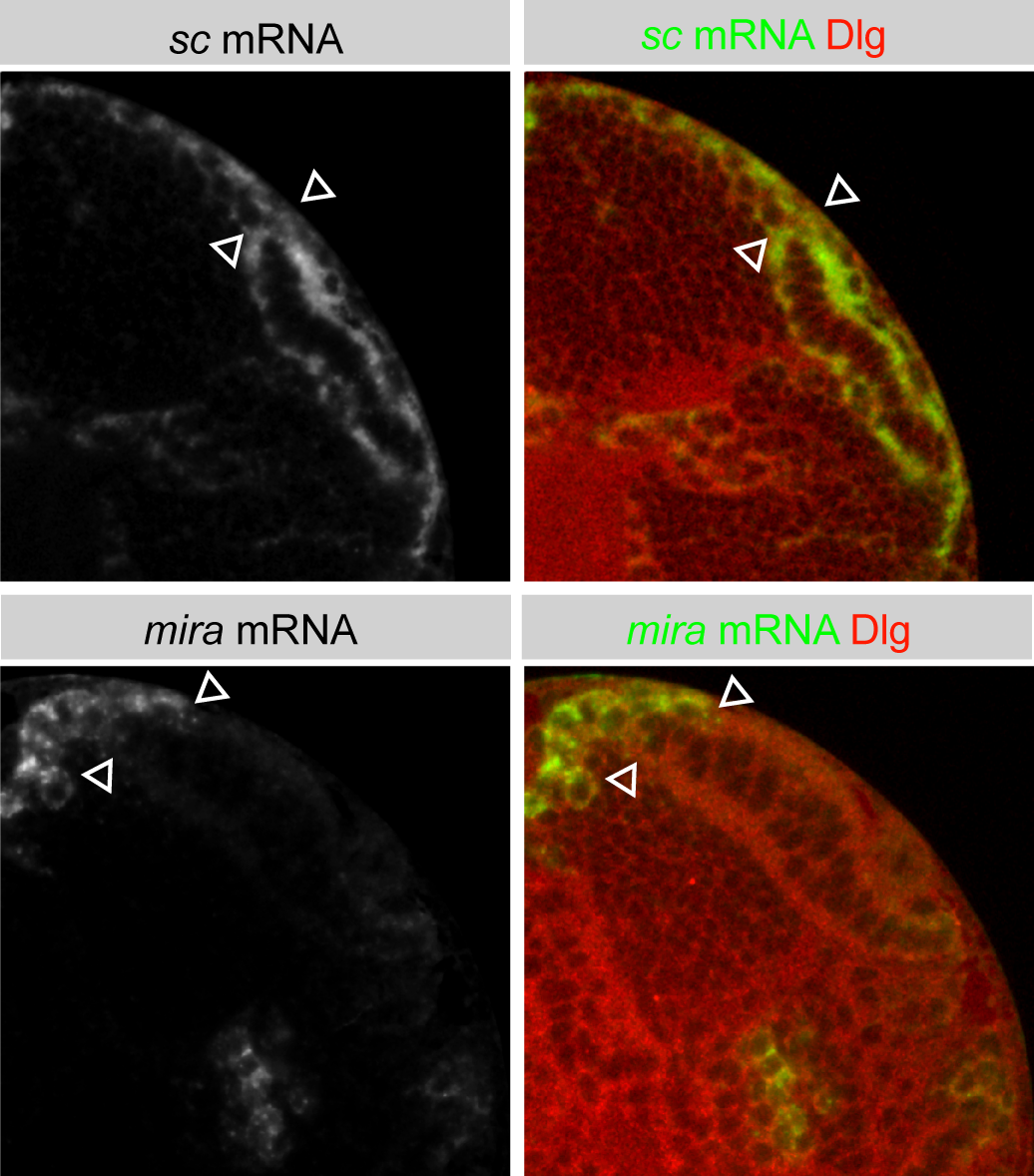

Supplement: Additional data file 1 — scute and mira mRNA expression in the optic lobe. (a) FISH to detect sc mRNA (green) in late third instar in combination with immuno-staining for Dlg (red). sc mRNA is expressed in the entire epithelium and in the medial neuroblasts. The 3.7sc-lacZ reporter line (Figure 2b) is expressed strongly in the neuroepithelium and is downregulated in medial neuroblasts. 3.7sc-lacZ may not reproduce the entire sc expression pattern, or lacZ expression in medial neuroblasts may be below our detection level. (b) FISH for mira mRNA (green) in late third instar in combination with immuno-staining for Dlg (red). mira mRNA is expressed in medial neuroblasts but not in neuroepithelial cells. [file 1749-8104-2-1-S1.png]
